# GPNMB Expression Associates with Inferior Prognosis in Patients with Small Cell Lung Cancer

**DOI:** 10.7150/jca.92661

**Published:** 2024-03-31

**Authors:** Qian Liu, Jun Zhang, Shiqi Mao, Dongdong Zhang, Youhong Dong, Pengchao Hu, Shengxiang Ren

**Affiliations:** 1 Department of Oncology, Xiangyang No. 1 People's Hospital, Hubei University of Medicine, Xiangyang, 441000, China.; 2 Department of Medical Oncology, Shanghai Pulmonary Hospital and Lung Cancer Institute, Tongji University School of Medicine, Shanghai, 200433, China.; 3 Department of Neurology, Xiangyang No. 1 People's Hospital, Hubei University of Medicine, Xiangyang, 441000, China.

**Keywords:** GPNMB, small cell lung cancer, metastasis, proliferation, prognosis

## Abstract

**Purpose**: Small cell lung cancer (SCLC) is widely recognized for its propensity for early and frequent metastases, which contribute to its status as a refractory malignancy. While the high expression of GPNMB in SCLC is well-documented, the precise correlation between GPNMB expression and the prognosis of SCLC remains undetermined.

**Methods:** HTG Edge-seq was used to screen the differential gene expression between primary SCLC lesions and paired metastatic lymph nodes (LN). The plasma concentration of GPNMB was measured using enzyme-linked immunosorbent assay (ELISA). The relationship between GPNMB concentration and clinical characteristics, as well as overall survival (OS) was assessed. One-to-one propensity score matching (PSM) was performed to reduce bias from confounding factors between groups. The invasive, migratory, proliferative, and apoptotic abilities of SCLC cells were evaluated using migration and matrigel invasion assays, CCK8 assay and flow cytometry respectively.

**Results**: GPNMB exhibited a significant up-regulation in LN compared to primary SCLC lesions as determined by HTG Edge-seq. Furthermore, patients with extensive disease demonstrated a significantly elevated plasma GPNMB concentration compared to those with local disease (*P* = 0.043). Additionally, patients with a high baseline plasma GPNMB level exhibited a shorter OS (10.32 vs. 16.10 months, *P* = 0.0299). Following PSM analysis, the statistical significance of the difference between the two groups persisted (9.43 vs. 15.27 months, *P* = 0.0146). Notably, both univariate and multivariate analyses confirmed that higher expression of GPNMB served as an independent biomarker for OS before PSM (*P* = 0.033, HR = 2.304) and after PSM (*P* = 0.003, HR = 6.190). Additionally, our study revealed that the inhibition of GPNMB expression through the use of siRNA effectively diminished the metastatic and proliferative capabilities of SCLC. Furthermore, this inhibition resulted in an enhanced ability to induce apoptosis.

**Conclusions:** In light of our findings, it can be inferred that the expression of GPNMB is linked to metastasis and an unfavorable prognosis, thus suggesting its potential as a novel therapeutic target in the treatment of SCLC.

## Introduction

Small cell lung cancer (SCLC) is recognized as the most aggressive variant of lung cancer, constituting approximately 15% of all lung cancer cases 1. A substantial majority, roughly two-thirds, of SCLC patients present with extensive disease upon initial diagnosis, resulting in a dismal 5-year survival rate of less than 1%. The malignancy and lethality of SCLC can be attributed to its remarkably aggressive biological features, particularly its heightened propensity for metastasis 2-4. Targeted therapy has emerged as a significant advancement in the treatment of advanced non-small cell lung cancer (NSCLC), leading to a notable extension in overall survival (OS) 5-7. Nevertheless, systemic chemotherapy remains the primary treatment modality for patients with SCLC, even in the era of immunotherapy 8. Consequently, there is an urgent need to identify adverse prognostic factors and develop targeted therapies to ameliorate the unfavorable prognosis observed in SCLC patients.

Glycoprotein nonmetastatic melanoma protein B (GPNMB), alternatively referred to as haematopoietic growth factor inducible neurokinin 1 (HGFIN) and dendritic cell associated heparin sulfate proteoglycan dependent integrin ligand (DC-HIL), is a type I transmembrane protein composed of 572 amino acids. It was initially cloned and characterized in 1995 9-11. GPNMB has demonstrated significant expression in malignant tissues and has been recognized as a promising therapeutic target in various types of cancer, including epithelial ovarian cancer, hepatocellular cancer, colorectal cancer, breast cancer, and NSCLC 12-17. Furthermore, the antibody-drug conjugate glembatumumab vedotin (GV), which specifically targets GPNMB, has shown initial efficacy in clinical trials for breast cancer, metastatic uveal melanoma, and osteosarcoma 18-20.

In our previous study, a total of 32 surgically resected primary tumors and LN metastases were collected for the purpose of screening metastasis-related genes in SCLC 21-23. Notably, the expression of GPNMB was found to be significantly higher in LN compared to primary lung cancer. Additionally, Li et al. have reported a prevalent overexpression of GPNMB in SCLC tumor tissues when compared to normal lung tissue 24. However, the potential of GPNMB in blood as a prognostic indicator for poor outcomes in SCLC patients, as well as its underlying biological mechanisms remain largely unknown. This study aimed to examine the correlation between plasma GPNMB levels and clinicopathological characteristics as well as prognosis in patients diagnosed with extensive stage small cell lung cancer (ES-SCLC). Additionally, we sought to investigate the impact of GPNMB on cell migration, invasion, proliferation and apoptosis in SCLC cell lines.

## Materials and Methods

### Patients and specimens

A total of 32 histologically confirmed SCLC patients with paired primary tumor tissue and matched LN metastases were included as previously described 21-23. The patients underwent surgical resection between 1978 and 2013 at the National Koranyi Institute of Pulmonology. Formalin-fixed, paraffin-embedded (FFPE) tissue samples were collected to identify differentially expressed genes. RNA expression analysis of 2,560 cancer-related genes was performed by HTG Edge-Seq targeted oncology biomarker panel as previously described 22.

The pathologically diagnosed SCLC patients at Xiangyang No. 1 People's Hospital of Hubei University of Medicine from 2017 to 2020 were retrospectively included. Electronic medical records were collected including, age, sex, ECOG PS, smoking history, comorbidities, staging and metastatic sites. The key inclusion criteria were: (1) patients of histopathologically diagnosed with SCLC; (2) patients with plasma specimens for ELISA testing; (3) patients whose Eastern Cooperative Oncology Group performance status was 0 - 2; and (4) patients who received etoposide plus carboplatin or cisplatin as first-line chemotherapy until disease progression or intolerance to chemotherapy. The key exclusion criteria were: (1) patients could not tolerate first-line chemotherapy; and (2) patients who had secondary malignant tumors. The data analysis deadline is June 5, 2021. OS was defined as the time interval between the date of diagnosis and the date of death or the last follow up. This study was approved by Xiangyang No. 1 People's Hospital Ethics Committee.

Blood samples were collected before the initiation of treatment with first line chemotherapy. Plasma was separated by centrifugation and then stored at -80°C until detection. ELISA assay was used to detect GPNMB expression level. Median GPNMB concentration (903.5 pg/mL) as cutoff value to analysis.

### Immunohistochemistry staining

GPNMB expression was stained with anti- human GPNMB monoclonal antibodies (Cat No. 66926-1-Ig, Proteintech) using a concentration at 1:500. After the recovery of antigen bubbled up in EDTA (Ethylene Diamine Tetraacetic Acid) for 8 min and inhibition of endogenous peroxidase activity for 30 min with 3% H_2_O_2_, the sections were incubated in primary antibody for 1 hour at RT (room temperature). Then sections were embedded in second antibody, an HRP Rabbit/Mouse immunoglobulins for 1 hour.

### Cell Lines and Cell Culture

Cell lines Beas-2B were established in shanghai pulmonary hospital. H446 were obtained as a gift from the Chinese Academy of Sciences Shanghai Branch Cell Bank (Shanghai, China). H196 were purchased from KeyGEN BioTECH (Nanjing, China). All cell lines were cultured according to American Type Culture Collection (ATCC) guidelines. Briefly, Beas-2B cells were cultured in DMEM (HyClone, USA) containing 5% fetal bovine serum (FBS, Gibco, USA). The SCLC cell lines were maintained 1640 medium (Gibco, USA) and were supplemented with 10% fetal bovine serum (Gibco, USA) and 1% penicillin and streptomycin (PS, Sigma, USA). All cells were incubated at 37°C in humidified air containing 5% CO_2_.

### Quantitative real-time PCR

The mRNA levels of GPNMB were detected by real-time PCR using SYBR Premix Ex Taq (TAKARA, Tokyo, Japan). The expression of β-actin was prepared as the negative control. Total RNA was extracted from untreated cells or from cells exposed to various treatments using Trizol reagent (TaKaRa, Shiga, Japan). Reverse transcription was performed using the RevertAid First Strand cDNA Synthesis Kit (Thermo scientific, Rockford, IL) according to the manufacturer's instructions, followed by quantitative real-time PCR (qRT-PCR) amplification of the cDNA (1 ng/mL concentration, and 2.5 ml per reaction). The sequences of RT primers for GPNMB are as follows: GPNMB forward: (5′-ACAAGGAATACAACCCAATA-3′), reverse: (5′-ATAGCCACTCCAGCACA-3′). β-actin: forward (5′-AAATCGTGCGTGACATTAA-3′), reverse (5′-CTCGTCATACTCCTGCTTG-3′). Gene expression levels were analyzed by ABI 7300 thermocycler (Applied Biosystems, Foster City, CA) using the following conditions: 1 cycle at 95°C for 20 min, 40 cycles at 95°C for 5 s and 60°C for 30 s. Data analysis was done using the 2^-ΔΔCt^ method for relative expression levels of the genes.

### siRNA Transfection

Transfections were performed for 48 hours on cells seeded in antibiotic-free medium using Lipofectamine RNAiMax (Invitrogen, USA) and OptiMEM (Gibco, USA) following manufacturer recommendations. Transfections were performed with non-targeting NC-GPNMB or GPNMB targeting siRNA (RIBIO, Guangzhou, China), The sequence of siRNA are as follows: genOFF™-st-h-GPNMB-001, sense:5′-GAAGAACGATCGAAATTCA-3′; genOFF™-st-h-GPNMB-002, sense: 5′-GGATAATACTGGCCTGTTT-3′.si-genOFF™-st-h-GPNMB-003, sense: 5′-CTAGCCACTTCCTCAATTA-3′. Percent of cells alive and total cell number were determined using the countess automated cell counter and tryphan blue (Invitrogen, USA) on H446 or H196 cells treated with NC-GPNMB or si-GPNMB after 48 hours transfection to determine viability and *in vitro* assays, interference efficiency was detected by qRT-PCR (Figure S1B, C).

### In-vitro migration and invasion assays

Cells were trypsinized and collected in respective 2% serum media and pelleted at 1000 rpm for 5 minutes. Cells were resuspended in respective 2% serum media and counted using the countess automated cell counter and tryphan blue (Invitrogen, Carlsbad, USA). For the transwell migration assay, 2 × 10^4^/200μl H446 or H196 cells were plated in the top chamber with the non-coated membrane (8 μm for 24-well plate; Corning Costar, NY, USA). For the invasion assay, 5×10^4^/200 μl H446 or H196 cells were plated in the top chamber with extracellular matrix gel (Corning, USA)-coated membranes (8 μm for 24-well plate; Corning Costar, NY, USA). In both assays, cells that did not migrate through or invade the pores were removed with a cotton swab. Cells on the lower surface of the membrane were fixed with methanol and stained with 0.1% crystal violet (Sigma, USA). Experiments were performed in three times. The invaded cells were photographed with an inverted phase-contrast microscope (Olympus, CKX41), and counted at least 3 randomly-selected images to represent the relative migration and invasion. H196 were counted by the average number of cells and H446 were counted by relative area per field of view.

### Cell proliferation analysis

The cell proliferation assay was performed using the CellCounting Kit-8 (CCK-8, Dojindo, Japan). A total of 3×10^3^ (H446) and 1.5×10^3^ (H196) treateded cells were seeded into 96-well plates. After incubating for 0-7 days, 10 μl CCK-8 solution was added to each well and then incubated for another 2 h at 37°C with 5% CO_2_. The optical density (OD value) was measured at a wavelength of 450 nm by scanning with a microplate reader (Thermo Fisher, Waltham, MA USA). The experiments were performed in triplicate.

### Cell-apoptosis assay

The rates of cell apoptosis were measured by an Annexin V-FITC/PI Apoptosis Detection Kit (CWbiotech, Beijing, China). According to the manufacturer's instructions, cells were harvested and washed with cold PBS twice. After being resuspended in 100 mL of binding buffer, cells were incubated with fluorescein isothiocyanate (FITC)-Annexin V and propidium iodide (PI) for 15 minutes in the dark. Then flow cytometry analysis of apoptosis was performed using BD FACS Calibur (BD Biosciences, San Jose, CA, USA). The results were analyzed with FlowJo (FlowJo, LLC, Ashland, OR, USA).

### Enzyme-linked immunosorbent assay (ELISA)

The levels of GPNMB in human plasma were measured by ELISA. Patient plasma samples were taken out of -80 °C fridge, then 100μl was added to each well and the measurement was carried according to the manufacturer's protocol (SAB, USA). The optical density (OD value) was measured at a wavelength of 450 nm by scanning with a microplate reader (Promega, USA). All the samples were tested in duplicate.

### Statistical analysis

Statistical analysis was carried out using SPSS software (version 27.0 for Mac) and GraphPad Prism software (Version 8 for Mac). In order to reduce bias from confounding factors between groups, one-to-one propensity score matching (PSM) was performed with a tolerance of 0.02. Matching covariates consisted of gender, age, PS score, smoking history, comorbidities, stage and tumor metastasis site. The Chi-square test or Fisher's exact test was used to analyze the association between GPNMB expression and clinicopathological parameters. The Kaplan-Meier method was used to analyze the survival probability and log-rank test was used to calculate the significance of differences. Cox proportional hazard model was applied for the univariate and multivariate analyses to calculate the hazard rations (HR) and 95% confidence intervals (95% CI). Parameters with the univariate P value of less than 0.1 were included in multivariate model. P values in this article were two sided and considered statistically significant when less than 0.05. Median GPNMB concentration (903.5 pg/mL) as cutoff value to analysis.

## Results

### Screen SCLC metastasis-associated genes

**Figure 1:**
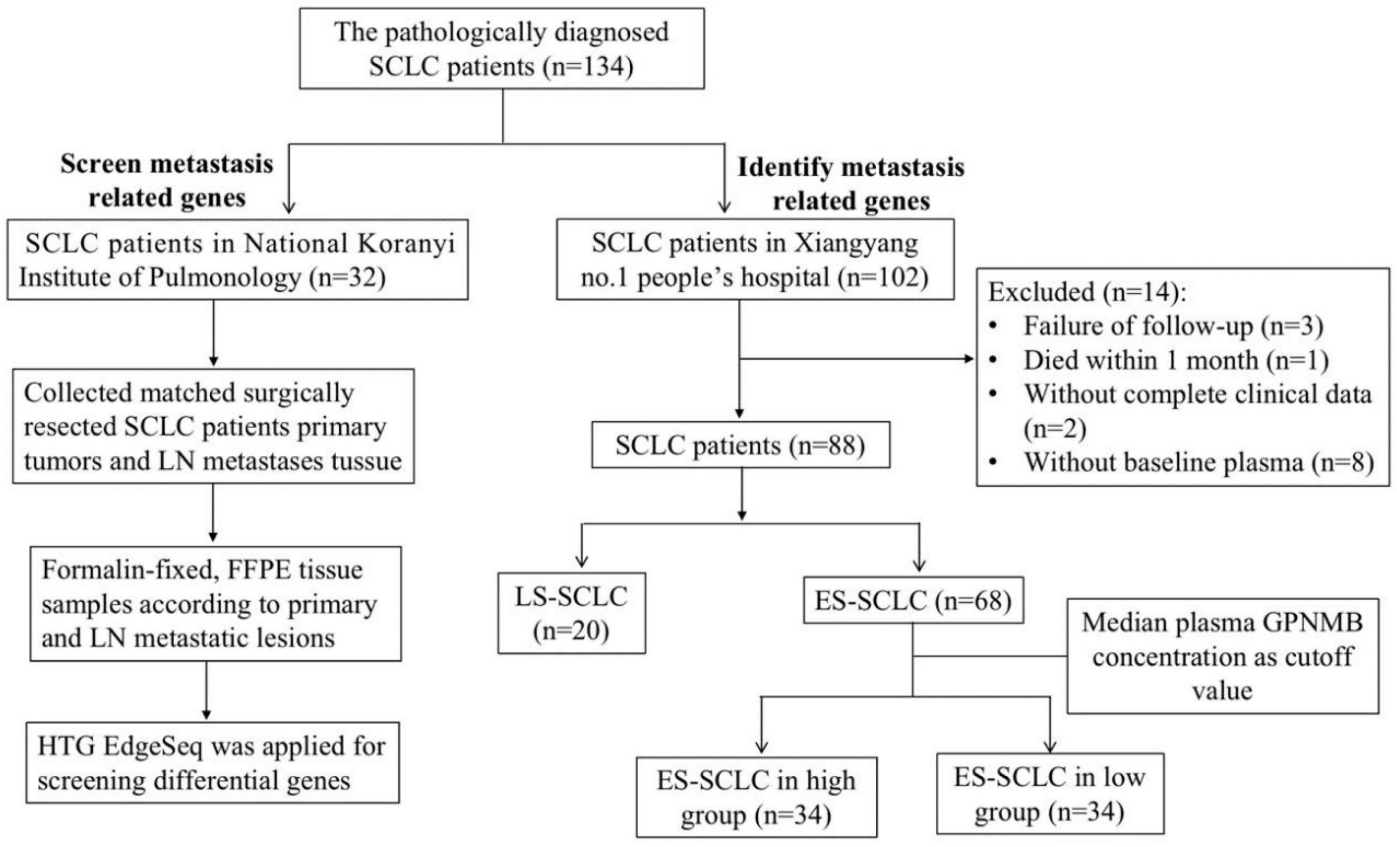
Flow chart of the study. 32 matched surgically resected SCLC primary tumors and LN metastases and 102 patients with plasma GPNMB concentration of SCLC in this study.

**Figure 2:**
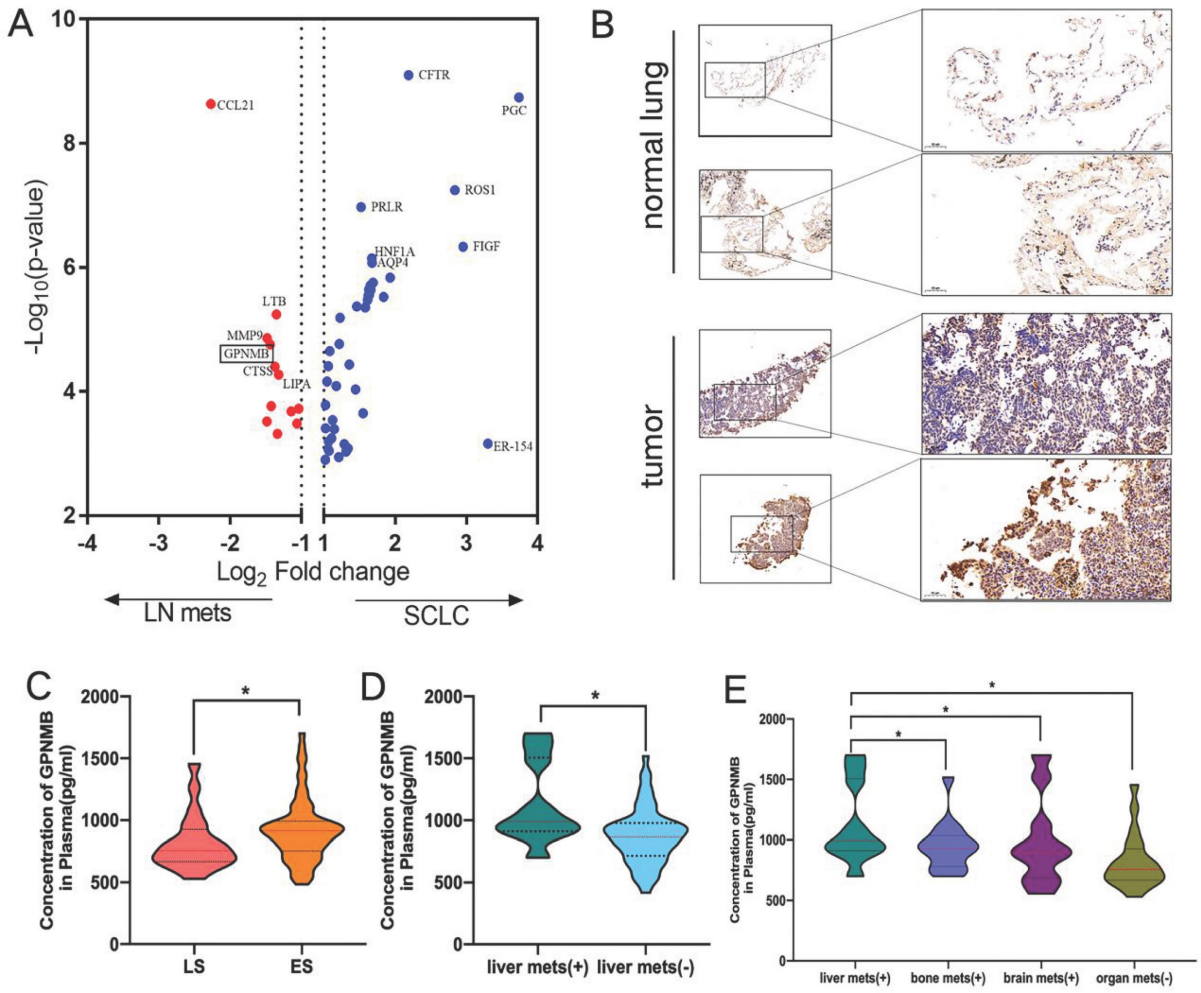
SCLC metastasis-associated gene analysis and higher concentration of GPNMB correlates with metastasis. (A) Differential gene expression in SCLC and metastasis LN by HTG edge-seq. (B) Representative figures of GPNMB staining in SCLC tumor and normal tissue. (C) Comparison of plasma GPNMB concentrations between LS (n=20) and ES (n=68) by ELISA. (D) Comparison of GPNMB concentrations between liver metastasis (n=10) and non-liver metastasis (n=58). (E) Comparison of GPNMB concentrations between liver metastases (n=10), bone metastases (n=14) and brain metastases (n=10) and non-distance metastasis (n=20). *P<0.05, **P<0.01, ***P<0.001 Statistical analysis was performed using student's t-test or Mann-Whitney U.

To screen for SCLC metastasis-related genes, we collected 32 matched surgically resected SCLC primary tumors and LN metastases in a previous study 21-23. We found 4 top significantly up-regulated genes in LN metastases by HTG Edge-seq (CCL21, LTB, MMP9, GPNMB) (Figure 2A). Except for genes that might have been highly expressed in the lymph nodes (CCL21, LTB) and has already previously identified related to tumor metastasis gene (MMP9), we selected GPNMB as target gene for further study. Subsequently, 15 SCLC tumor tissue and 6 normal lung samples were collected from Xiangyang No. 1 Peoples' Hospital and subjected to immunohistochemical analysis to assess GPNMB expression. Our results revealed a heightened level of GPNMB expression in SCLC tissues compared to that in normal lung samples (Figure 2B). Then we collected 102 pathologically diagnosed SCLC patients' clinical data and plasma from Xiangyang No. 1 People's Hospital, excluded some patients, a total of 88 patients of SCLC were further analyzed in this study (Figure 1).

### Comparison of GPNMB in SCLC patients and clinical characteristics

The average plasma GPNMB concentration in SCLC was 889.7 pg/mL (range: 417.0-1702.4 pg/mL, median: 863.7 pg/mL). The majority of patients were ES-SCLC (68, 77.3%). Then the baseline GPNMB concentration was compared. It was found that the average plasma GPNMB concentration in ES-SCLC group (916.6 pg/mL) was markedly higher than LS-SCLC (755.9 pg/mL) (*P* = 0.0435) (Figuer 2C). We further found that average plasma GPNMB concentration in liver metastasis group was significantly higher than non-liver metastasis (P = 0.0148) (Figure 2D). Moreover, we found that the plasma GPNMB concentration in liver metastasis were particularly higher than bone metastasis (P = 0.031), brain metastasis (P = 0.035) and non-organ metastasis (P = 0.005) (Figure 2E). However, there were no significant difference in bone metastasis and brain metastasis when compared with non-distant organs metastasis group (P > 0.05) (Figure S2).

Then we evaluated the relationships between GPNMB concentration and baseline clinical characteristics in ES-SCLC patients. Patient characteristics in ES-SCLC are summarized in Table 1. The median age was 64.5 years (range: 43-79). 22 patients (32.4%) had never smoked, and most of the patients had good performance status (95.6%) and non-comordities (76.5%). The most common metastatic site was bone (20.6%). Median GPNMB concentration (903.5 pg/mL) as cutoff value equally divided into two groups. Higher GPNMB concentration groups positively correlated with liver metastasis (P=0.040), while it is similar regarding to different T stage (P=0.230), N stage (P=0.134), M stage (P=0.595), brain metastasis (P=0.493) and bone metastasis (P=0.230) (Table 1). As shown in Table 1, higher GPNMB concentration groups were more likely to liver metastasis. As a result of PSM balancing the differences in characteristics, 17 pairs of patients were analyzed. The difference between the two groups was not significant (Table 1).

### Association of plasma GPNMB concentration with clinical outcome

**Figure 3:**
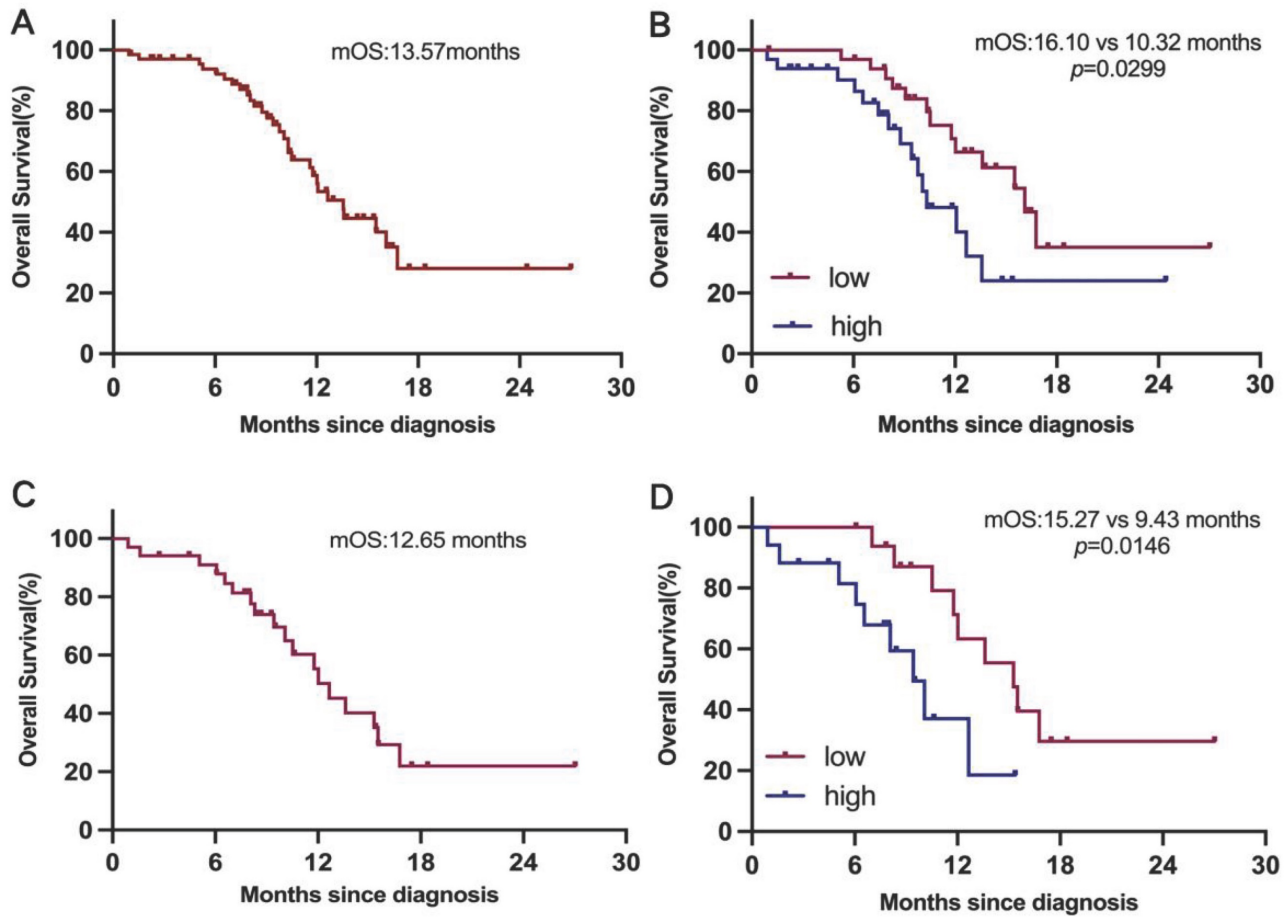
Higher concentration of GPNMB correlates with dismal overall survival before and after PSM. (A) Kaplan-Meier curves for OS in overall (n=68). (B) Kaplan-Meier curves for OS based on GPNMB concentration. Median GPNMB concentration (903.5 pg/mL) as cutoff value divided into high group (n=34) and low group (n=34). (C) Kaplan-Meier curves for OS in overall after PSM (n=34). (D) Kaplan-Meier curves for OS based on GPNMB concentration after PSM. Differences were assessed using the log rank (Mantel-Cox) test.

We next investigate the clinical relevance of GPNMB concentration in 68 ES-SCLC patients. Survival analysis indicated that median OS was 13.57 months (Figure 3A). Patients in high GPNMB concentration group had a markedly shorter median OS than those in low GPNMB concentration group (10.32 vs. 16.10 months, *P* = 0.0299) (Figure 3B). Following the application of propensity score matching, median OS was determined to be 12.65 months (Figure 3C). Notably, patients belonging to the high GPNMB concentration group had a markedly shorter median OS than those in low GPNMB concentration group (9.43 vs. 15.27 months, *P* = 0.0146) (Figure 3D).

The univariate analysis for OS suggested that comorbidities (P = 0.022, HR = 2.708), liver metastasis (*P* = 0.004, HR = 4.536), more metastasis sites (*P* = 0.034, HR = 2.676) and high level of GPNMB (*P* = 0.021, HR = 2.440) were associated with shorter median OS. The multivariate analysis showed that GPNMB concentration (*P* = 0.033, HR = 2.304) was the remaining independently factors for inferior OS. After PSM analysis, the multivariate analysis also revealed that GPNMB concentration was the remaining independently factors for inferior OS (*P* = 0.003, HR = 6.19). The detailed information of univariate and multivariate analysis is shown in Table 2.

### GPNMB silencing inhibits invasion and migration of SCLC

**Figure 4:**
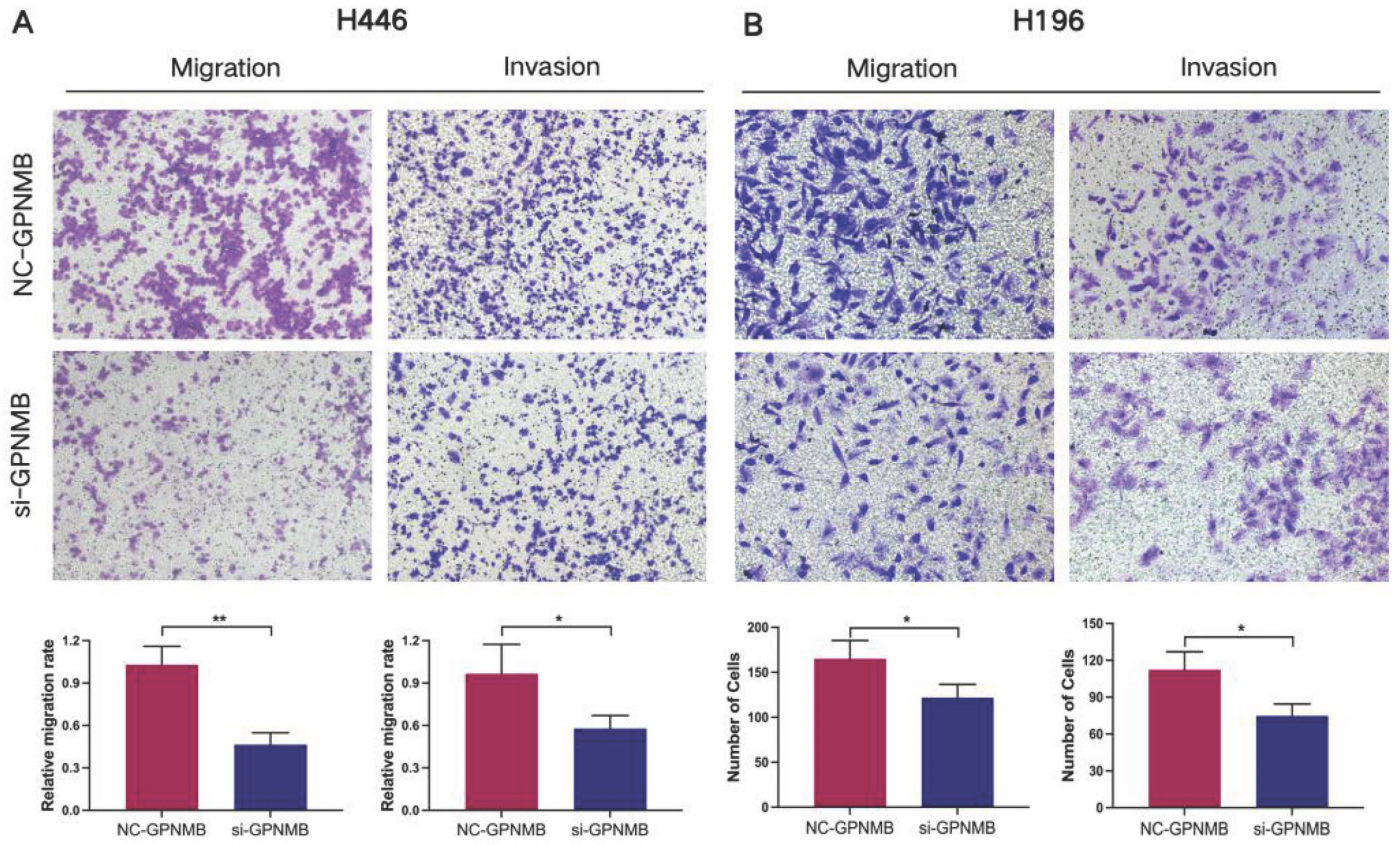
GPNMB promotes SCLC cells migration and invasion *in vitro*. Transwell assay was used to determine the migration and invasion ability of SCLC cells. H446 cells and H196 cells treated with NC-GPNMB or si-GPNMB for 24 h. (magnification, 10×). *P<0.05, **P<0.01 and statistical analysis was performed using student's t-test.

Next, we explored the functions of GPNMB on SCLC cells *in vitro*. The expression of GPNMB in SCLC cell lines (H446, H196) was significantly higher than that in normal bronchial epithelial cell line (Beas-2b) in RNA levels (Figure S1A). Then we checked migration and invasion in SCLC cell lines. In H446 and H196 cells, GPNMB were downregulated by small interfere RNA compared with respective controls. Interference efficiency was detected by qRT-PCR (Figure S1B, C). We found that GPNMB knockdown significantly inhibited the migration and invasion of H446 and H196 cells (Figure 4). Collectively, these results demonstrate that GPNMB can enhance the migratory and invasive abilities in SCLC.

### GPNMB silencing inhibits proliferation and increase apoptosis of SCLC

**Figure 5:**
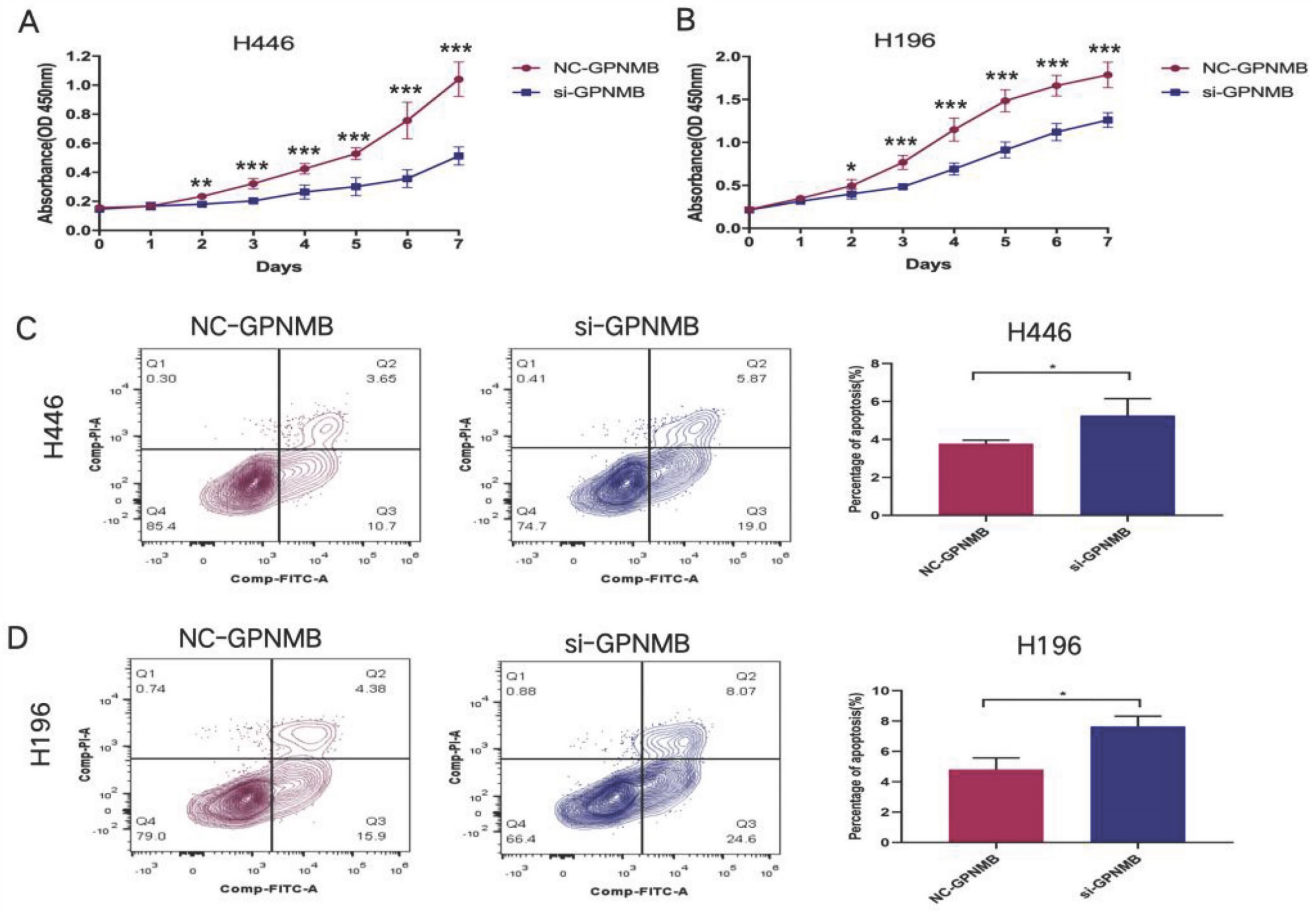
GPNMB promotes SCLC cells proliferation and inhibits apoptosis *in vitro*. (A, B) A total of 3×10^3^ (H446) and 1.5×10^3^ (H196) treated cells were seeded into 96-well plates. After incubating for 0-7 days, 10 μl CCK-8 solution was added to each well and then incubated for another 2 h at 37 °C. The optical density (OD value) was measured at a wavelength of 450 nm Growth curves of cells were measured by CCK-8 assays in H446 and H196 cells. (C, D) A total of 5 × 10^5^ (H446) and 2 × 10^5^ (H196) treated cells were seeded into 6-well plates incubating for 48 hours. Representative flow cytometry images showed the apoptosis of H446 and H196 cells. Data are represented as mean ± SEM. *P<0.05, **P<0.01, ***P<0.001 and statistical analysis was performed using student's t-test.

Additionally, we explored the proliferation and apoptosis functions of GPNMB on SCLC cells *in vitro*. CCK8 assay showed that knockdown of GPNMB attenuated cell proliferation in H446 and H196(Figure 5A, B). Furthermore, downregulation of GPNMB promote SCLC cells apoptosis *in vitro* (Figure 5C, D). These results indicated that GPNMB may promote the proliferation and inhibit apoptosis of SCLC.

## Discussion

In the current investigation, GPNMB upregulation was observed in LN metastatic lesions compared to primary tumors using HTG Edge-seq. Initially, we discovered a significant elevation in plasma GPNMB expression among ES-SCLC patients relative to LS-SCLC patients. Moreover, a higher concentration of GPNMB was associated with a poorer clinical prognosis in ES-SCLC patients. Additionally, our findings indicate that GPNMB plays an important role in promoting cancer cell migration and invasion.

GPNMB is a transmembrane glycoprotein that exhibits expression in various cancer types and is particularly highly expressed in the majority of cancerous tissue, indicating an unfavorable prognosis. Consequently, GPNMB has been investigated as a potential therapeutic target for malignancies 10. Li et al. demonstrated a significant association between GPNMB and N stage in patients diagnosed with head and neck squamous cell carcinoma (HNSCC), with elevated GPNMB expression levels in HNSCC being linked to a poor prognosis 25.

It is worth noting that liquid biopsy, a non-invasive diagnostic method, possesses distinctive advantages and plays a crucial role in guiding precision diagnosis and treatment of malignant tumors 26-28. In this study, we initially assessed the expression of plasma GPNMB in patients diagnosed with SCLC. The results indicated a significant elevation of GPNMB expression in patients with ES-SCLC compared to those with LS-SCLC. Additionally, we observed higher levels of plasma GPNMB concentration in patients with liver metastasis compared to those with metastasis in non-distant organs. Furthermore, patients belonging to the high plasma GPNMB concentration group exhibited a significantly poorer OS compared to those in the low GPNMB group. Previous investigations utilizing whole-exome sequencing analysis have also reported higher GPNMB expression in patients of colorectal cancer (CRC) with liver metastasis than paired primary CRC, and its association with poor prognosis 29. Other Similar results have indicated that GPNMB is a negative prognostic indicator in human colorectal liver metastasis 30. Building upon this, our findings suggest that GPNMB plays a role in the progression of SCLC and may potentially serve as a predictive marker for metastasis and unfavorable outcomes.

Subsequently, we conducted *in vitro* cell experiments to investigate the specific functions of GPNMB and its underlying pathogenesis. Prior investigations have documented the role of GPNMB in activating mutated EGFR signaling and facilitating metastasis 16. Moreover, other studies have indicated that GPNMB fosters invasion, migration, and metastasis of lung cancer cells 17, albeit these findings were predominantly based on NSCLC. It is widely recognized that SCLC exhibits distinct biological characteristics compared to NSCLC. Our study is firstly to reveal that GPNMB was upregulated in SCLC cell lines, and downregulation of GPNMB hindered migration, invasion, and proliferation while enhancing apoptosis capabilities of SCLC cells. GPNMB has been implicated in the promotion of cancer cell metastasis through multiple mechanisms. Prior research has identified GPNMB as a crucial molecular mediator that facilitates the acquisition of aggressive traits by inducing matrix metalloproteinases (MMPs) and epithelial-mesenchymal transition (EMT) 31-33. Additionally, GPNMB, known for its role as a negative regulator of T cell activation, exhibits potent immunosuppressive properties in the context of cancer 11. A previous study has demonstrated that the suppression of GPNMB expression in Tumor endothelial cells leads to T-cell exhaustion, implying that Tumor endothelial cells (TECs) may facilitate the evasion of cancer cells from immune surveillance via GPNMB 34. Likewise, GPNMB can enhance the proliferation and spread of melanoma and propel tumor advancement by means of an immunosuppressive mechanism that hinders T cell activation 35. Additionally, the upregulation of GPNMB expression can induce adaptive resistance in anti-PD-1 therapy by re-establishing immunosuppression 36. Consequently, it is imperative to conduct further investigation into the biological impacts of GPNMB on SCLC immune regulation.

Glembatumumab vedotin (CDX-011, GV) is a fully human Immunoglobulin G2 monoclonal antibody that specifically targets GPNMB and is linked to monomethyl auristatin E (MMAE), a potent cytotoxic microtubule inhibitor 20. Currently, a series of clinical trials are underway to investigate the efficacy of GV in cancer therapy, and these trials have shown promising preliminary results 18-20, 37, 38. In a phase II study focusing on patients with metastatic uveal melanoma, the overall response rate, safety, and survival were evaluated. The findings indicate that GV was well-tolerated, and the disease control rate remained high and sustained 20. In a similar vein, the phase II study aimed to examine the efficacy of GV in advanced breast cancer through the assessment of GPNMB expression. In comparison to chemotherapy, GV demonstrated favorable tolerability 18. Another study discovered that HSP90 inhibitors induce GPNMB cell-surface expression, thereby enhancing the sensitivity of breast cancer cells to GV 39. Presently, there is a lack of effective targeted medication for SCLC, necessitating further investigation into the potential applicability of GV in this context.

It is imperative to acknowledge the presence of several limitations in this study. Firstly, the restricted sample size necessitates a larger sample size in future research endeavors to thoroughly examine the characteristics of GPNMB in SCLC patients. Additionally, while we have demonstrated the promotion of metastasis and proliferation in SCLC cells by GPNMB, further investigation is required to elucidate the effects of GPNMB on the immune microenvironment and the role of GV in SCLC.

In summary, our study revealed a significant correlation between elevated expression of plasma GPNMB and unfavorable prognosis as well as liver metastasis in a clinical context. Furthermore, our findings indicate that GPNMB has the potential to enhance invasion, migration, and proliferation in SCLC cells. These results suggest that GPNMB holds promise as a promising therapeutic target for individuals diagnosed with SCLC in the foreseeable future.

## Supplementary Material

Supplementary figures.

## Figures and Tables

**Table 1:** Correlations between GPNMB concentration and clinical characteristics before and after propensity score matching.

Characteristics		Unadjusted analysis				Propensity score matching			
		Total (N=68) (%)	Low (N=34) (%)	High (N=34) (%)	*P* value	Total (N=34) (%)	Low (N=17) (%)	High (N=17) (%)	*P* value
Age									
	≥65 years	34 (50.0)	16 (47.1)	19 (55.9)	0.467	16 (47.1)	6 (35.5)	10 (58.8)	0.169
	<65 years	34 (50.0)	18 (52.9)	15 (44.1)		18 (52.9)	11 (64.7)	7 (41.1)	
Gender									
	Male	63 (92.6)	33 (97.1)	30 (88.2)	0.356	32 (94.1)	16 (94.1)	16 (94.1)	1.000
	Female	5 (7.4)	1 (2.9)	4 (11.8)		2 (5.9)	1 (5.9)	1 (5.9)	
ECOG PS									
	0~1	65 (95.6)	32 (94.1)	33 (97.1)	1.000	33 (97.1)	17 (94.1)	16 (94.1)	1.000
	2	3 (4.4)	2 (5.9)	1 (2.9)		1 (2.9)	0 (0.0)	1 (5.9)	
Smoking history									
	never smoker	22 (32.4)	10 (29.4)	12 (35.3)	0.604	11 (32.4)	6 (35.3)	5 (32.4)	0.714
	former/current smoker	46 (67.6)	24 (70.6)	22 (64.7)		23 (67.6)	11 (64.7)	12 (70.6)	
Comorbidities									
	Yes	16 (23.5)	6 (17.6)	10 (29.4)	0.253	8 (23.5)	3 (17.6)	5 (29.4)	0.688
	No	52 (76.5)	28 (82.4)	24 (70.6)		26 (76.5)	14 (82.4)	12 (70.6)	
T stage									
	1-2	14 (20.6)	9 (26.5)	5 (14.7)	0.230	6 (17.6)	2 (11.8)	4 (23.5)	0.656
	3-4	54 (79.4)	25 (73.5)	29 (85.3)		28 (82.4)	15 (88.2)	13 (76.5)	
N stage									
	0-2	26 (38.2)	10 (29.4)	16 (47.1)	0.134	15 (44.1)	8 (47.1)	7 (41.2)	0.730
	3	42 (61.8)	24 (70.6)	18 (52.9)		19 (55.9)	9 (52.9)	10 (58.8)	
M stage									
	0	20 (29.4)	11 (32.4)	9 (26.5)	0.595	11 (32.4)	6 (35.3)	5 (29.4)	0.714
	1	48 (70.6)	23 (67.6)	25 (73.5)		23 (67.6)	11 (64.7)	23 (67.6)	
Baseline brain metastasis									
	No	58 (85.3)	28 (82.4)	30 (88.2)	0.493	28 (82.4)	13 (76.5)	15 (88.2)	0.656
	Yes	10 (14.7)	6 (17.6)	4 (11.8)		6 (17.6)	4 (23.5)	2 (11.8)	
Baseline bone metastasis									
	No	54 (79.4)	29 (85.3)	25 (73.5)	0.230	30 (88.2)	15 (88.2)	15 (88.2)	1.000
	Yes	14 (20.6)	5 (14.7)	9 (26.5)		4 (11.8)	2 (11.8)	2 (11.8)	
Baseline liver metastasis									
	No	58 (85.3)	32 (94.1)	26 (76.5)	**0.040**	29 (85.3)	15 (88.2)	14 (82.4)	1.000
	Yes	10 (14.7)	2 (5.9)	8 (23.5)		5 (14.7)	2 (11.8)	3 (17.6)	

**Table 2:** Univariate and multivariate analyses of clinical parameters on OS before and after propensity score matching.

Factors	Unadjusted analysis						Propensity score matching					
	Univariate analyses			Multivariate analyses			Univariate analyses			Multivariate analyses		
	HR	95% CI	P value	HR	95% CI	P value	HR	95% CI	P value	HR	95% CI	P value
Age (≥65 years/<65 years)	1.912	0.873-4.188	0.105				2.297	0.826-6.389	0.111			
Gender (Male/Female)	0.426	0.127-1.421	0.165				0.820	0.107-6.28	0.849			
ECOS PS (2/0~1)	3.793	0.489-29.393	0.202				1.000	0-1.091E5	1.000			
Smoking history (ever/ never)	1.376	0.625-3.031	0.428				0.928	0.327-2.483	0.882			
Comorbidities (Yes/ No)	2.708	1.153-6.363	**0.022**	2.071	0.871-4.927	0.099	4.009	1.474-10.906	**0.007**	4.296	1.231-14.994	**0.022**
Brain metastasis (Yes/ No)	1.306	0.494-3.456	0.590				2.100	0.567-7.780	0.267			
Bone metastasis (Yes/ No)	0.809	0.280-2.336	0.695				4.330	1.162-16.138	**0.029**	0.925	0.153-7.628	0.938
Liver metastasis (Yes/ No)	4.536	1.609-12.789	**0.004**	3.232	0.846-12.339	0.086	2.655	0.710-9.927	0.147			
Number of metastatic sites (0/1-3)	2.676	1.080-6.634	**0.034**	1.353	0.411-4.452	0.619	3.674	1.216-11.105	**0.021**	4.540	1.042-19.774	**0.044**
GPNMB concentration (High/ Low)	2.440	1.147-5.194	**0.021**	2.304	1.072-4.952	**0.033**	3.181	1.127-8.979	**0.029**	6.19	1.823-21.019	**0.003**

## Abbreviations

SCLC: Small cell lung cancer
LS-SCLC: limited stage small cell lung cancer
ES-SCLC: extensive stage small cell lung cancer
GPNMB: Glycoprotein nonmetastatic melanoma protein B
ELISA: enzyme-linked immunosorbent assay
GV: glembatumumab vedotin
OS: overall survival
LN: lymph node
ECOG PS: Eastern Cooperative Oncology Group performance status
T: tumor
N: node
M: metastasis
HR: hazard rations
CI: confidence interval
MMPs: matrix metalloproteinases
EMT: epithelial-mesenchymal transition
TECs: Tumor endothelial cells
MMAE: monomethyl auristatin E
