# Supplementary material for: GPNMB Expression Associates with Inferior Prognosis in Patients with Small Cell Lung Cancer
Source: J Cancer. 2024 Mar 31;15(10):2960–70. doi: 10.7150/jca.92661 (PMC11064273; doi:10.7150/jca.92661)
Supplement: Supplementary file 1 — Supplementary figures. [file jcav15p2960s1.pdf]

Supplementary figures

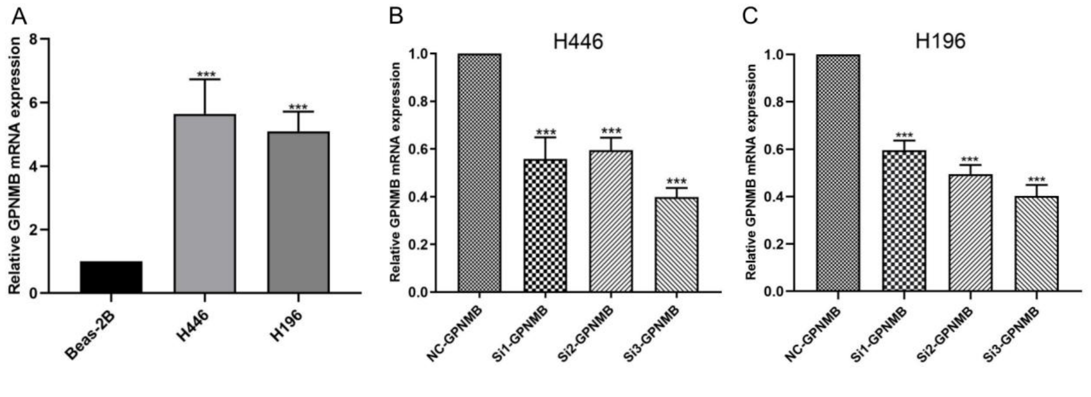


Fig.S1 GPNMB upregulated in SCLC cells and promotes SCLC cells migration, invasion, proliferation and inhibits apoptosis by EMT in vitro. (A, B) mRNA and protein expression levels of GPNMB in SCLC cell lines (H446, H196) and normal bronchial epithelial cells (Beas-2b). (C-F) The silence efficiency of GPNMB in H446 and H196 were confirmed by qRT-PCR and western blot. Data are presented as mean ± SD from three independent experiments. Transfections were performed for 48 hours on SCLC cells. *P<0.05, **P<0.01, ***P<0.001 Statistical analysis was performed using student's t-test.

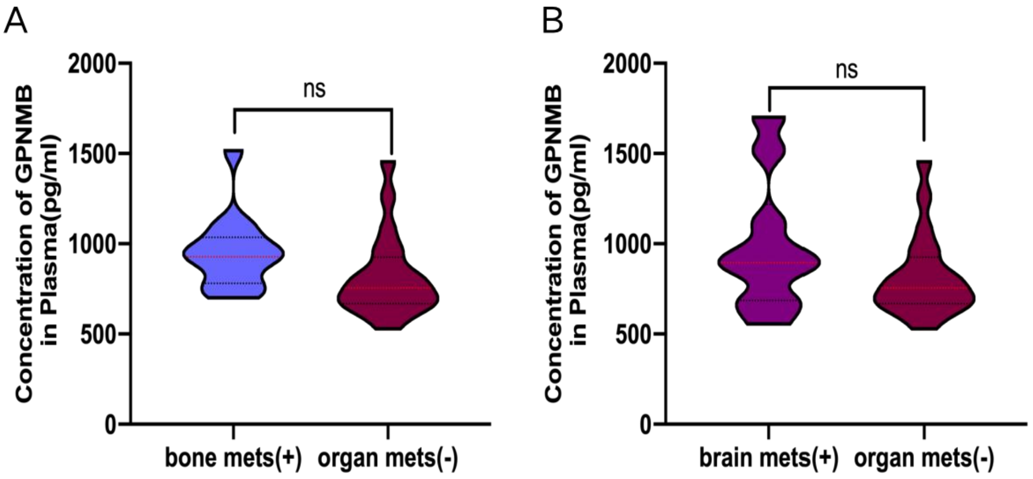


Fig.S2 (A) Comparison of GPNMB concentrations between bone metastasis and non-distance metastasis. (B) Comparison of GPNMB concentrations between brain metastasis and non-distance metastasis. ns, not significant $*P < 0.05$, $**P < 0.01$, $***P < 0.001$ and Statistical analysis was performed using student's t-test or Mann-Whitney U.
